# An Optimal Hierarchical Decision Model for a Regional Logistics Network with Environmental Impact Consideration

**DOI:** 10.1155/2014/542548

**Published:** 2014-03-17

**Authors:** Dezhi Zhang, Shuangyan Li, Jin Qin

**Affiliations:** ^1^School of Traffic & Transportation Engineering, Central South University, Changsha, Hunan 410075, China; ^2^College of Transportation and Logistics, Central South University of Forestry and Technology, Changsha, Hunan 410004, China

## Abstract

This paper proposes a new model of simultaneous optimization of three-level logistics decisions, for logistics authorities, logistics operators, and logistics users, for regional logistics network with environmental impact consideration. The proposed model addresses the interaction among the three logistics players in a complete competitive logistics service market with CO_2_ emission charges. We also explicitly incorporate the impacts of the scale economics of the logistics park and the logistics users' demand elasticity into the model. The logistics authorities aim to maximize the total social welfare of the system, considering the demand of green logistics development by two different methods: optimal location of logistics nodes and charging a CO_2_ emission tax. Logistics operators are assumed to compete with logistics service fare and frequency, while logistics users minimize their own perceived logistics disutility given logistics operators' service fare and frequency. A heuristic algorithm based on the multinomial logit model is presented for the three-level decision model, and a numerical example is given to illustrate the above optimal model and its algorithm. The proposed model provides a useful tool for modeling competitive logistics services and evaluating logistics policies at the strategic level.

## 1. Introduction

Environmental issues have recently attracted considerable public attention around the world. There is a wide consensus that freight transportation is a major contributor to climate change and global warming is due to various pollution emissions. It has been shown that freight transportation contributes to about 5.5% of global greenhouse gas emissions [1]. The report on CO_2_ emissions in 25 European countries during 1990–2005 also indicated that about 26% of total CO_2_ emissions in the air come from transportation. During logistics service activities, the CO_2_ emissions from transportation amount to 93% of total pollution emissions, while the warehousing only covers 7% [2]. It is, therefore, very important and urgent to create an environmentally sustainable logistics system.

Green logistics focus on improvement of logistics service efficiency, decrease in logistics cost, and reduction in environmental externalities (e.g., CO_2_) so as to achieve a sustainable balance among economic, environmental, and social objectives [3, 4]. Aronsson [5] showed that logistics efficiency and cost were not only related to the structure of supply chains, but also related to the logistics network design and logistics infrastructure. As an important component of a regional logistics system (or urban logistics system), logistics network design is a strategic issue, involving logistics facility planning and sustainable logistics management policy-making [6, 7].

Various logistics centers have recently been established in large cities for quickly distributing freight. However, this raises many important issues, such as traffic congestion, air pollution, and high energy consumption. To efficiently cope with these issues, it is proposed to combine multiple logistics centers into a logistics park. A logistics park, which is also referred to as “logistics village” in Germany, “distribution park” in Japan, and “logistics platform” in Spain, is a specially important component of the regional logistics network. A logistics park implies a spatial concentration area for grouping various activities, such as transportation, distribution, warehousing, commercial trade, and other related services (such as maintenance and repair). It is also an intersection of different transport modes and an interface between local traffic and long-distance traffic [22, 23].

Logistics parks play an important role in environmental effects (e.g., reducing CO_2_ emissions and air pollution) in Germany [24]. Owing to the successful practices of logistics park operations in Germany and Japan, there is a growing trend in introducing logistics parks in some developing countries. For example, in China, there are increasingly logistics park projects under construction or in planning, from 207 in 2006, to 475 in 2008, up to 754 in 2012, according to the survey data by the China Federation of Logistics & Purchasing [25]. However, there are some important problems in the planning and operation of the logistics park projects in China. For example, logistics users' behavioral responses to the project investment were seldom considered in the design of logistics parks, leading to a low usage rate of the logistics parks. Since the construction of a logistics park consumes a great deal of money and land, the number and size of the logistics parks should be carefully designed.

There are a number of studies on logistics network design in the literature. They can be classified into two major classes in terms of modeling methodology: Hub-Spoke location models based on classical location theory and network equilibrium models based on spatial price equilibrium theory. As far as the Hub-Spoke location models are concerned, O'Kelly [26] formulated the hub location problem as a quadratic integer programming model. Sender and Clausen [10] presented a network model of wagonload traffic, which aimed to determine the hub location and size considering total cost and efficiency of the network system. Alumur et al. [11] investigated a hub location problem from the perspective of network design, jointly considering the transportation cost and travel time and proposed a mixed integer programming formulation. Tang et al. [27] presented an optimization model for the location planning problem of logistics parks with variable capacity. The goal of their model was to determine the optimal locations and to allocate the customers to the logistics parks using a hybrid heuristic algorithm. Crainic et al. [28] addressed a two-tiered freight distribution system in a big city. They presented a location-routing model to determine the optimal locations of logistics facilities (primary facilities and secondary facilities) and optimal sizes and routes for different vehicle fleets.

As far as the network equilibrium modeling approach is concerned, Friesz et al. [29] presented some important freight network models for predicting intercity freight movement and provided a case study about the U.S. coal industry. Harker and Friesz [14] reviewed the major modeling techniques that had been applied in analyzing intercity freight network equilibrium and pointed out the shortcomings of the spatial price equilibrium models. Taniguchi et al. [30] proposed a bi-level model to determine the optimal size and location of public logistics terminals and solved the model using queuing theory and nonlinear programming techniques. Nagurney [31] investigated a multimodal multicommodity problem from the perspective of supply chain equilibrium and obtained a Nash-Cournot equilibrium solution based on variational inequality theory. Yamada et al. [19] presented a network equilibrium model to integrate supply chain networks with transportation networks, taking into account the behavior of freight carriers. Bauer et al. [21] addressed a multicommodity, capacitated intermodal freight transportation network planning problem, which considered greenhouse gas emissions as the primary objective. Up to now, the freight network equilibrium modeling approach has been widely applied in practical logistics network design [17, 18, 32–35].

Table 1 further summarizes the previous related studies on logistics network design problems. It indicates that the existing related studies mainly focused on minimizing transportation cost or time, and little attention has been paid to environment-related costs and the effects of economies of scale. “Economies of scale” refer to the phenomenon that the average construction cost per unit area for a logistics park and the average operating cost per unit of shipment decrease with the number and size of logistics parks. It is of great importance to consider these effects in the design of the logistics parks particularly in an era of (capital and land) resource shortage and climate change.

**Figure 1:**
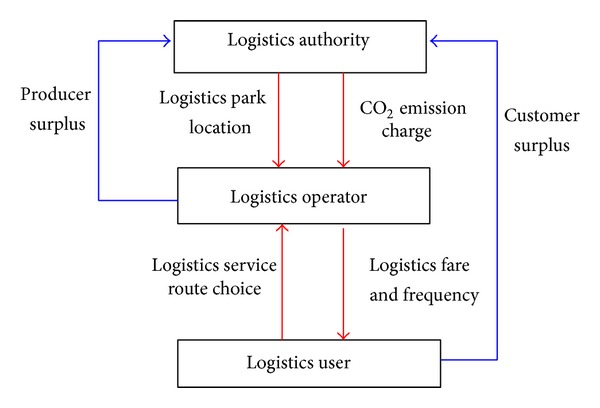
Relationships among the different players in logistics network.

In view of the above, our goal in this research is to investigate the interaction among the three logistics players in a complete competitive logistics service market considering the location of logistics park and CO_2_ emission charge. To this end, we propose a three-level logistics decisions model on green regional logistics network considering the environmental impact as shown Figure 1.

In this multilevel decision system, the logistics authority aims to maximize the total social welfare of the system (i.e., the sum of customer surplus and producer surplus), by two different methods: optimizing the location of logistics parks and applying the CO_2_ emission charge.

Logistics operators are assumed to compete with logistics service fares, service time, and service delay, while logistics users minimize their own perceived logistics disutility given the logistics service fare and frequency by logistics operators.

The remainder of this paper is organized as follows.  Section 2 describes regional logistics network components and decision-making behaviors.  Section 3 provides the optimal decision model and corresponding algorithms.  Section 4 presents a numerical case study and discusses the result.  Section 5 concludes the paper.

## 2. Basic Considerations

### 2.1. Network Representation

**Figure 2:**
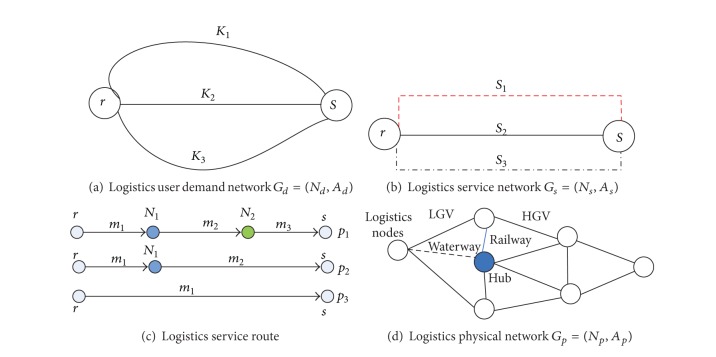
Network representation of the regional logistics system.

In order to model the regional logistics services, Figures 2(a)–2(d) show the logistics demand network, logistics service network, service route, and physical logistics network, respectively. In Figure 2(a), there are different types of logistics demands (such as industrial, commercial, and agricultural logistics) between a given logistics origin-destination (OD) pair. These demands are served by the logistics service network, as shown in Figures 2(b) and 2(c).

Let *G*_*s*_ = (*N*_*s*_, *A*_*s*_) denote the logistics service network (such as pick-up/delivery, storage, and transfer). Let *N*_*s*_ represent a set of nodes in which logistics activities are implemented. A service, denoted as *a*_*s*_ ∈ *A*_*s*_, is defined by a logistics service route in the physical logistics network *G*_*p*_ = (*N*_*p*_, *A*_*p*_). It consists of a sequence of logistics nodes on the service route and is differentiated by type of logistics services (such as transportation mode, service cost, and service time). A logistics leg is a nonstop component of a logistics service route that is defined by itinerary, service cost and time, and transportation mode.

The logistics physical network, as shown in Figure 2(d), is composed of a set of logistics nodes (logistics parks, distribution centers, and freight terminals) and a set of logistics links or arcs which represent physical links, such as road segments, rail tracks, river segments, or sea lines. We denote a logistics physical network as *G*_*p*_ = (*N*_*p*_, *A*_*p*_), where *N*_*p*_ is the set of logistics nodes (or transfer nodes) and *A*_*p*_ is the set of logistics links. All transfers take place at logistics transfer nodes. In this paper, two different types of logistics transfer nodes are considered: one is the logistics park with economy of scale effects and the other is a general transfer node with a small capacity, such as a distribution center.

**Figure 3:**
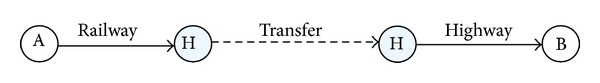
Representation of virtual transfer arc.

In order to facilitate the construction of the model, we introduce a virtual arc to represent a logistics transfer activity (i.e., change of transportation modes). For example, Figure 3 represents a logistics service from node A to B, via transferring from railway to highway at transfer node H. In this paper, for presentation purpose, we denote *N*_*s*_^*h*^ ⊂ *N*_*s*_ as the set of logistics transfer nodes and *A*_*s*_^*h*^ ⊂ *A*_*s*_ as the set of virtual logistics transfer arcs.

### 2.2. Description of Decision Questions in This Paper

**Figure 4:**
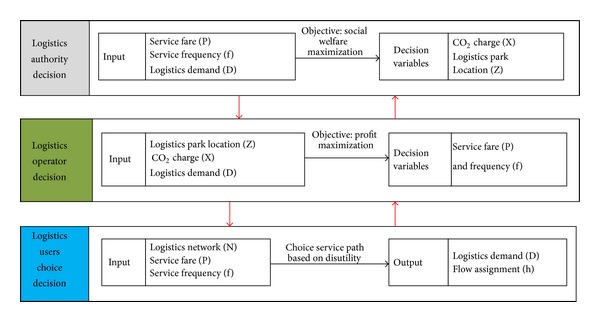
Decision relationship analyses on regional logistics network.

As Figure 4 shows, the logistics authority (government) implements its optimal objective (i.e., maximum of social welfares) by optimizing location of logistics park and CO_2_ emission charge strategy, so as to affect the logistic operators and logistics users' behaviors.

Logistics operators choose their optimal logistics service frequency based on the given logistics network structure and logistics management policy. The logistics users make their logistics path choice decisions based on their own perceived expected logistics service disutility.

## 3. Model Formulation and Solution Analysis

### 3.1. Assumptions

To facilitate the presentation of the essential ideas without loss of generality, the following basic assumptions are made in this paper.

*A1*. The planning period is assumed to be one week. The model proposed in this paper is thus mainly used for strategic planning and/or policy evaluation purposes.

*A2*. In the regional logistics system, the locations of logistics parks and CO_2_ emission taxes are determined by the logistics authority. 

*A3*. Logistics users make their logistics service route choices based on their own perceptions on the service disutility. The disutility of logistics service is measured by service time and fare. 

*A4*. An elastic demand function is used to capture the responses of logistics users to the disutility of logistics service. 

*A5*. The logistics users can use a single (pure) mode or a combination of several modes (called combined mode). The single/pure mode includes heavy goods vehicle (HGV), light goods vehicle (LGV), railway, or waterway. 

*A6.* A service arc presents a logistics operator. And logistics operators maximize their individual profit by competing with logistics service fare and delivery frequency.

### 3.2. Notations

*Sets*

 
*M*: set of all transport modes in regional logistics service market, *M* = *M* ^*s*^ ∪ *M* ^*c*^
 
*M* ^*s*^: set of pure transport modes; “1”, “2”, “3”, and “4”, respectively, represent the HGV, LGV, railway, and waterway
 
*M* ^*c*^: set of combined transport modes
 
*W*: set of all origin-destination (O-D) pairs in the logistics network
 
*R*_*w*_ = *R*_*w*_^*s*^ ∪ *R*_*w*_^*c*^: set of all service routes between OD pair *w* ∈ *W*
 
*R*_*w*_^*s*^, *s* ∈ *M* ^*s*^: set of routes by pure mode *s*
 
*R*_*w*_^*c*^, *c* ∈ *M* ^*c*^: set of routes by combined mode *c*
 
*R*_*w*,*i*_^*c*^, *c* ∈ *M* ^*c*^: set of routes by combined service mode *c* via transfer node *i*
 
*I*: set of transfer nodes
 
**S**_*i*_ = {*s*_*i*_^1^, *s*_*i*_^2^,…}: set of alternative sizes of candidate logistics park.

*Variables Associated with Logistics Users*

 
*q*_*w*_: realized demand between OD pair *w* ∈ *W* (tons/week)
 
*q*_*w*_^*m*^: realized demand between OD pair *w*, *w* ∈ *W*, serviced by transport mode *m*, *m* ∈ *M* (tons/week)
 
*q*_*w*,*i*_^*c*^: logistics demand between OD pair *w* ∈ *W* via transfer node *i* ∈ *I* (tons/week)
 
*λ*_*w*_: expected minimal disutility between OD pair *w* ∈ *W* ($)
 
*u*_*wr*_^*m*^: disutility on route *r* ∈ *R*_*w*_ between OD pair *w* ∈ *W* by transport mode *m* ($)
 
*h*_*wr*_^*m*^, *m* ∈ *M*: freight flow served by combined transport mode *m* on route *r* ∈ *R*_*w*_ between OD pair *w* ∈ *W* ($)
 
*h*_*wr*_^*s*^, *s* ∈ *M* ^*s*^: freight flow served by pure transport mode *s* on route *r* ∈ *R*_*w*_^*s*^ between OD pair *w* ∈ *W* (tons/week)
 
*h*_*wr*_^*c*^, *c* ∈ *M* ^*c*^: freight flow served by combined transport mode *c* on route *r* ∈ *R*_*w*_^*c*^ between OD pair *w* ∈ *W* (tons/week)
 
*h*_*wr*,*i*_^*c*^: freight flow on route *r* ∈ *R*_*w*_^*c*^ via transfer node *i* between OD pair *w* ∈ *W* (tons/week)
 
*v*_*a*_^*m*^: freight flow serviced by transport mode *m* on logistics service arc *a* ∈ *A*_*s*_^*M*^ (tons/week)
 
*δ*_*ar*_^*m*^: equals 1 if link *a* is on route *r* by mode *m*, and 0 otherwise.

*Variables Associated with Logistics Operators*

 
*p*_*a*_^*m*^: fare of logistics service by operator *m* over arc *a* ($/ton-km)
 
*f*_*a*_^*m*^: frequency of logistics service by operator *m* over arc *a* (shifts/week)
 
*S*_*a*_^*m*^: service capacity per shift of operator *m* over arc *a* (tons/shift).

*Variables Associated with Logistics Authority*

 
*x*_*a*_^*m*^: 0-1 variable, equal to 1 if the vehicle *m* over arc is charged and 0 otherwise
 
*X* = (*x*_*a*_^*m*^): vector of the 0-1 variable *x*_*a*_^*m*^;
 
*e*: emission taxes per unit CO_2_ emission ($/kg)
 
*z*_*a*_^*m*^: 0-1 variable, equal to 1 if the potential logistics transfer node *i* is chosen as the logistics park and 0 otherwise
 
*Z* = (*z*_*a*_^*m*^): vector of the 0-1 variable *z*_*a*_.

*Constants*

 
${\overset{-}{q}}_{w}$: potential demand between OD pair *w* ∈ *W* (tons/week)
 
*t*_*a*_^*m*^: average service time on arc *a* ∈ *A*_*s*_^*M*^ by transport mode *m* (hour)
 
*l*_*a*_: length of arc *a* ∈ *A*_*s*_^*M*^(km)
 
*c*_*a*_^*m*^: variable costs of unit shipment on arc *a* ∈ *A*_*s*_^*M*^ served by mode *m* ($/ton-km)
 
*τ*_*a*_^*m*^: fixed operator cost per trip on arc *a* ∈ *A*_*s*_^*M*^ served by mode *m* ($/round)
 Cap_*a*_^*m*^: service capacity on arc *a* ∈ *A*_*s*_^*M*^ by mode *m* (tons/km)
 Cap_*i*_: service capacity at transfer node *i* ∈ *I* (tons/week)
 
*c*_*i*_: unit transfer fare charged at logistics node *i* ∈ *I* ($/ton)
 
*C*_*i*_^*o*^: unit construction cost (fixed cost) at logistics node *i* ∈ *I*($/m^2^)
 
*η*_*i*_: unit transfer operating cost at logistics node *i* ∈ *I* ($/ton)
 
*ρ*: parameter for capturing the effects of economies of scale for logistics parks
 
*e* ^*m*^: average CO_2_ emission per unit turnover by transport mode *m* ∈ *M* (kg/ton-km).

### 3.3. Logistics Users' Service Choice Equilibrium

According to A3, the logistics users' disutility consists of the logistics service time and transportation cost, which can be expressed as

(1) $$\begin{array}{c} u_{wr}^{m}=C_{wr}^{m}+\tau_{\text{vot}}T_{wr}^{m},\;\forall r\in R_{w}^{},\;\;w\in W,\;\;m\in M, \end{array}$$

where *C*_*wr*_^*m*^, *T*_*wr*_^*m*^, and *D*_*wr*_^*m*^ represent the transportation cost, logistics service time, and CO_2_ emission taxes on service route *r* by transport mode *m* between OD pair *w*, respectively. *τ*_vot_ is the value of time.

The transportation cost and service time on a route can be expressed as the sum of the transportation costs and service times on all the arcs along that route, including transfer cost and time, which are, respectively, expressed as

(2) $$\begin{array}{c} C_{wr}^{m}=\underset{a\in A_{s}}{\sum }c_{a}^{m}\delta_{ar}^{m},\;\forall r\in R_{w}^{},\;w\in W,\;m\in M, \end{array}$$

(3) $$\begin{array}{c} T_{wr}^{m}=\underset{a\in A_{s}}{\sum }t_{a}^{m}\left(v_{a}^{m}\right)\delta_{ar}^{m},\;\forall r\in R_{w}^{},\;\;w\in W^{},\;\;m\in M. \end{array}$$

Considering the difference in the attributes of different transportation modes, the logistics service time *t*_*a*_^*m*^(*v*_*a*_^*m*^) on link *a* for different modes should be estimated by different functions, as shown in (4). For the transportation mode HGV or LGV, the Bureau of Public Roads US (BPR) type function can be used to estimate the service time [36]. For railway or waterway, the average in-vehicle travel time and departure interval time should be considered; that is,

(4) tam(vam)={tam0(1+0.15(vamCapam)4),m=1,2tam0+tmdmax⁡(vam−Capam,0)Capam,m=3,4 ∀a∈As,

where *t*_*a*_^*m*0^ is the free-flow travel time and *t*_*md*_ is the average shift interval for transport mode *m*.

According to A3, the logistics service mode/route choices can be calculated by a logit-based stochastic user equilibrium (SUE). The flow *h*_*wr*_^*m*^ on route *r* between OD pair *w* ∈ *W* served by mode *m* can thus be given by

(5) hwrm=qwexp⁡(−θuwrm)∑m∈M∑r∈Rwexp⁡(−θuwrm),∀r∈Rw,w∈W,m∈M,

where the parameter *θ* represents the variation of perception of logistics users on logistics service disutility, which can be calibrated by using the survey data. The higher the value of *θis*, the smaller the variation in logistics users' perception will be, and vice versa.

In order to capture the logistics users' responses to logistics service disutility, an elastic travel demand function is introduced. It is assumed that the total resultant demand *q*_*w*_ between OD pair *w* is a continuous and monotonically decreasing function, *D*_*w*_(·), of the expected minimal service disutility *λ*_*w*_ for the logistics OD pair concerned [37]; that is,

(6) $$\begin{array}{c} q_{w}^{}=D_{w}\left(\lambda_{w}^{}\right),\;\forall w\in W. \end{array}$$

In this paper, we adopt the following demand function:

(7) $$\begin{array}{c} q_{w}={\overset{-}{q}}_{w}\exp\left(-\beta \lambda_{w}\right), \end{array}$$

where ${\overset{-}{q}}_{w}$ is the potential logistics service demand between OD pair *w* and *β* is the demand dispersion parameter that reflects the demand sensitivity to the logistics service disutility by OD pair *w*. According to random utility theory, *λ*_*w*_ can be measured by the following log-sum formula [20, 38]:

(8) $$\begin{array}{c} \lambda_{w}=-\frac{1}{\theta }\ln\left(\underset{m\in M}{\sum }\;\underset{r\in R_{w}^{}}{\sum }\exp\left(-\theta \mu_{wr}^{m}\right)\right). \end{array}$$

The network equilibrium for a multimodal logistics network with elastic demand can be defined as follows.

**Definition 1**

A network flow pattern (*h*_*wr*_^*s*^, *h*_*wr*_^*c*^, *h*_*wr*,*i*_^*c*^, *q*_*w*_^*m*^, *q*_*w*_) is a stochastic user equilibrium (SUE) for a multimodal logistics network with elastic demand if it satisfies (1)–(8).

**Algorithm 2 (Algorithm for the model of logistics user's service choice)**

Therefore, once the logistics service frequencies and logistics fares of all operators are given, the logistics demand distribution over all of the logistics service sub-networks can then be determined. The algorithm is given as follows.

**Step 1 (initialization)**

Set iteration index *n* = 1. Let *v*_*a*_^(0)^ = 0, for  all  *a* ∈ *A*, and set the logistics service time and fare under the free-flow conditions for all links and compute the route logistics service cost and time {*C*_*wr*_^*m*^, *T*_*wr*_^*m*^, for  all  *r* ∈ *R*_*w*_, *w* ∈ *W*, *m* ∈ *M*} according to (2), (3), and (4).

**Step 2 (calculating the disutility)**

Calculate the disutility of all logistics service routes *u*_*wr*_^*m*^ according to (1). Compute the expected minimum disutility, *λ*_*w*_, between logistics OD pair *w* based on (8).

**Step 3 (calculating logistics demand)**

Calculate the resultant demand ${\overset{-}{q}}_{w}^{\left(n\right)}$ for each OD pair *w* based on (7).

**Step 4 (logit-based SUE assignment)**

Conduct logit-based SUE assignment to get auxiliary link flow *g*_*a*_^(*n*)^, *a* ∈ *A*; the auxiliary flows at logistics nodes (i.e., virtual transfer links) are computed by *g*_*i*_^(*n*)^ = ∑_*w*∈*W*_∑_*r*∈*R*_ *h*_*wr*_^*m*(*n*)^.

**Step 5 (method of successive average)**

Let *v*_*a*_^(*n*+1)^ = *v*_*a*_^(*n*)^ + (1/(*n* + 1))(*g*_*a*_^(*n*)^ − *v*_*a*_^(*n*)^), *a* ∈ *A*.

**Step 6 (convergence check)**

Define $\mathrm{gap}(n)=\sqrt{\sum_{a\in A}^{}{\left(v_{a}^{\left(n+1\right)}-v_{a}^{\left(n\right)}\right)}^{2}}/\sum_{a\in A}^{}v_{a}^{\left(n\right)}$, and if gap⁡(*n*) ≤ *ξ*, then go to Step 8 and output all results as the final solution, where *ξ* is a predetermined parameter.

**Step 7**

Set *n* = *n* + 1, compute all link travel costs, and route travel costs on the basis of current link flows and then go to Step 2.

**Step 8**

Output the resultant demand and corresponding network flow pattern *h* = (*h*_*wr*_^*s*^, *h*_*wr*_^*c*^, *h*_*wr*,*i*_^*c*^, *q*_*w*_^*m*^, *q*_*w*_).

### 3.4. Logistics Operator Service Frequency and Fare Submodel

Logistics operators compete with each other by optimizing their own service frequency and logistics fares. This process can be modeled as a noncooperative Nash game. At equilibrium, no logistics operator has an incentive to deviate or change his/her decision variables given all other logistics operators' decisions. To formulate the competitive equilibrium between logistics operators, the net profit of a logistics operator is first defined as follows:

(9) Φm(ym,y−m)=∑apamvam−∑avamτam −∑aCamfam−∑avamlaxame,

where *y*_^*a*^_^*m*^ = (*p*_*a*_^*m*^, *f*_*a*_^*m*^) is the fare and frequency of logistics operator *m* over link *a*, **y**_  _^*m*^ = (*y*_1_^*m*^,…, *y*_*k*_^*m*^) is the vector of the fare and frequency of operator *m*, and **y**_  _^−*m*^ = (*y*_1_^−*m*^,…, *y*_*k*_^−*m*^) is that of the other operators excluding *m*.

Equation (9) is the logistics operator *m* profit function, in which the first part is the revenue, the second part is the variable operation cost associated with freight flow, the third part is the frequency-related cost, and the last one is the cost of CO_2_ emission tax.

With the assumption of Cournot-Nash's competition game among logistics operators, a logistics operator's profit maximization problem is formulated as follows:

(10) $$\begin{array}{c} \underset{\left\{y^{m}\right\}}{\max}\Phi_{m}\left(y^{m},y^{-m}\right),\;\forall m\in M, \end{array}$$

subject to

(11) $$\begin{array}{c} v_{a}^{m}\leq S_{a}^{m}f_{a}^{m},\;\forall a\in A_{s}^{M},\;m\in M, \end{array}$$

(12) $$\begin{array}{c} \underset{m}{\sum }v_{a}^{m}\leq \max\left\{\text{t}{\text{r}}_{a}^{0}\left(1-Z_{a}\right),\text{t}{\text{r}}_{{\;}_{a}}^{1}Z_{a}\right\},\;\forall a\in A_{s}^{H}. \end{array}$$

Constraint (11) indicates that the total logistics flow serviced by logistics operators on arc *a* of subnetwork *G*_*s*_^*m*^ must not exceed the maximum logistics service capacity (in terms of tons/week). Constraint (12) states that the total logistics flow must not exceed the capacity of processing of the transfer logistics nodes, tr_*a*_^0^, and tr_*a*_^1^ represents the transfer capacity of the traditional transfer node and logistics park, respectively, *z*_*a*_ = {0,1} and 1 means the node is logistics park and otherwise for traditional transfer node.

In this paper, a Lagrangian relaxation approach and a penalty function approach are adopted to incorporate these side constraints into the objective function (10) [39]. The augmented Lagrangian penalty function for logistics operator *m* is constructed as

(13) Lm(ym,τ,ξ)=πm(ym,y−m) −12ρ[∑a∈AsM(max2{0,τa+ρ(vam−Samfam)}−τa2)    +∑a∈AsH(max2{0,ξa+ρ(∑m∈Mvam                  −max⁡{φa0(1−Za),                  φa1Za})}        −ξa2)],

where *ρ* is a penalty constant; *τ*_*a*_^*k*^ and *ξ*_*a*_ are the Lagrange multipliers associated with side constraints (11)-(12), respectively; and *τ*, *ξ* are the corresponding vectors. The capacitated maximization problem (9)–(12) is thus equivalent to the following unconstrained maximization problem:

(14) $$\begin{array}{c} \underset{\left\{y^{m}\right\}}{\max}\;L_{m}\left(y^{m},\tau ,\xi \right),\;\forall m\in M. \end{array}$$

The equilibrium solutions for the service frequencies and fares of logistics service for operator *m* can be determined by the resultant unconstrained maximization problem (14). This can be solved by a heuristic solution algorithm that combines the diagonalization method and the Hooke-Jeeves method [40]. The basic idea of the algorithm is to solve the individual unconstrained problem (14) for each logistics operator separately and sequentially using the Hooke-Jeeves method, holding the decision variables of the other logistics operators fixed in turn until the sequence converges.

**Step 1 (initialization)**

Choose an initial fare and frequency pattern *y* ^(1)^ = {*p* ^(1)^, *f* ^(1)^} for all *m* logistics operators, initial Lagrange multipliers *τ* ^(1)^ = {*τ*_*a*_^*k*(1)^}, *ξ* ^(1)^ = {*ξ*_*a*_^(1)^}, and a positive penalty parameter *ρ* ^(1)^. Set the iteration counter to *n* = 1.

**Step 2 (determination of logistics user assignment flow pattern)**

Determine the resultant logistics demand *q*_*w*_^(*n*)^ and route flow pattern *h* ^(*n*)^ for each logistics service subnetwork using (1)–(10).

**Step 3 (diagonalization)**

Solve the unconstrained maximization problems as (14) separately and sequentially for *m* logistics operator using the Hooke-Jeeves method and then generate the auxiliary fare and frequency pattern ${\overset{-}{y}}^{\left(n\right)}=\left\{{\overset{-}{p}}^{\left(n\right)},{\overset{-}{f}}^{\left(n\right)}\right\}$.

**Step 4 (updating)**

Update the fare and frequency pattern: $y^{\left(n+1\right)}=y^{\left(n\right)}+\left({\overset{-}{y}}^{\left(n\right)}-y^{\left(n\right)}\right)/\left(n+1\right)$.

**Step 5 (termination check)**

If all of the side constraints (11)-(12) are satisfied, then terminate and output the optimal solution y* = *y* ^(*n*+1)^; otherwise go to Step 6.

**Step 6 (multipliers and penalty constant updating)**

Update the multipliers *τ* and *ξ* by

(15) τa(n+1)=max⁡{0,τa(n)+ρ(n)(vam−Samfam)}, ∀a∈As,ξa(n+1)=max⁡{0,ξa(n)     + ρ(n)(∑m∈Mvam−max⁡⁡{φa0(1−Za),φa1Za})},∀a∈AsH

and update the penalty constant by

(16) $$\begin{array}{c} \rho^{\left(n+1\right)}=\{\begin{array}{cc} k\rho^{\left(n\right)}, & \text{if}\sqrt{\sigma^{\left(n+1\right)}+\upsilon^{\left(n+1\right)}}>\gamma \sqrt{\sigma^{\left(n\right)}+\upsilon^{\left(n\right)}},\\ \rho^{\left(n\right)}, & \text{otherwise}, \end{array} \end{array}$$

where

(17) σ(n)=∑a∈Asmax2{−τa(n)ρ(n),(vam−Samfam)},υ(n)=∑a∈AsHmax2{−ξa(n)ρ(n),      ∑m∈Mvam−max⁡{φa0(1−Za),φa1Za}}.

Set the parameters *γ* = 0.25 and 2 ≤ *k* ≤ 10, as recommended by Bertsekas [41]. Set *n* = *n* + 1 and go to Step 2.

In Step 3, *m* unconstrained maximization problems need to be solved independently using the Hooke-Jeeves method [42], one for each logistics operator until all logistics operators' best responses are obtained. It should be pointed out that the order of solving each problem may affect the final result. Consequently, random orders of cycles need to be generated in the solution process and then the “best” solution from various logistics demands which maximizes the social welfare of the system defined in the next subsection is chosen as the optimal solution.

### 3.5. Logistics Authority Submodel

As previously stated, the logistics authority aims to determine the optimal logistics parks location and CO_2_ charge policy in order to maximize the total social welfare.

SW is the total social welfare per unit period, defined as the sum of consumers' surplus and logistics operators' profit; that is,

(18) max⁡ X,ZSW=(∑w∈W∫0qwDw−1(w)dw−∑w∈Wqwλw) +(∑i∈Icihi−∑i∈ICi0(yi)ρxi−∑i∈Iηihi) +∑m∈MΦm

(19) $$\begin{array}{c} \underset{a\in A_{s}^{H}}{\sum }Z_{a}\leq p \end{array}$$

(20) $$\begin{array}{c} Z_{a}^{}=\left\{0,1\right\},\;\forall a\in A_{s}^{H}, \end{array}$$

(21) $$\begin{array}{c} x_{a}^{m}=\left\{0,1\right\},\;\forall a\in A_{s}^{},\;m\in M, \end{array}$$

where the first part of the right hand of (18) represents consumers' surplus, while the second and third ones are the profit of transfer operators and transportation operators, respectively, which are both producer's surplus; *D*_*w*_^−1^(·) is the inverse function of logistics demand, which is relative to logistics user disutility and a continuous, monotonically decreasing function.

Constraint (19) indicates that the maximal number of logistics parks to construct is *p*, and constraints (20) and (21) express a binary variable for location for logistics parks and CO_2_ emission charges, respectively.

### 3.6. Analysis of Solution for the Optimization Model

Let *Ω* be the set of decisions of government *Ω* = {(*X*_*a*_^(*i*)^, *Z*_*b*_^(*i*)^), for  all  *i* = 1,2, 3,…, *a* ∈ *A*_*s*_^*M*^, *b* ∈ *A*_*s*_^*H*^}.

Suppose there is a countable decision plan on the logistics park location and the CO_2_ emission charge for the government to decide, and we denote the *i*th decision plan (*X*_*a*_^(*i*)^, *Z*_*b*_^(*i*)^).

**Step 1 (initialization)**

Set $\underline{\text{SW}}=-\infty$ (i.e., the lower bound of the objective function SW in (18)). Choose an initial feasible plan for the government decision set *Ω*.

**Step 2 (first loop operation (logistics authority implicit enumeration loop))**

Perform a complete logistics authority decision in decision set *Ω*. Set the logistics authority decision plan counter to *i* = 1.

**Step 3 (second loop operation (logistics operators loop))**

Set the logistics operator counter to *j* = 1. Given the logistics authority decision plan (*X*_*a*_^(*i*)^, *Z*_*b*_^(*i*)^), solve the logistics operator service frequency and fare submodel (10)–(12) using the solution algorithm that is proposed in Section 3.4 to obtain the optimal logistics service fare and frequency pattern **y** ^(*j*)^ = (**p** ^(*j*)^, **f** ^(*j*)^).

**Step 4**

If >|*M*|, then go to Step 7.

**Step 5 (third loop operation (logistics service path flow assignment loop))**

Given the logistics authority decision plan (*X*_*a*_^(*i*)^, *Z*_*b*_^(*i*)^) and logistics operator decision vector **y** ^(*j*)^ = (**p** ^(*j*)^, **f** ^(*j*)^), solve the logistics service choice submodel (1)–(8) using the solution algorithm that is proposed in Section 3.3 to obtain the corresponding logistics demand *q*__*w*__^(*t*)^ and logistics service route flow *h*_  _^(*t*)^ = {*h*_*wr*_^*m*(*t*)^}, and the logistics service disutility *u*_  _^(*t*)^ = {*u*_*wr*_^*m*(*t*)^}. And then calculate the objective function value *SW*_  _^(*i*)^ for the current decision plan (*X*_*a*_^(*i*)^, *Z*_*b*_^(*i*)^).

**Step 6 (termination check for the second loop)**

If $W_{{\;}_{}}^{\left(j\right)}\geq \underline{\text{SW}}$, then put y* = **y** ^(*j*)^,  h* = *h* ^(*j*)^, SW* = *SW* ^(*i*)^, *j* = *j* + 1, and go to Step 3. Otherwise, set *j* = *j* + 1 and go to Step 3.

**Step 7 (termination check for the first loop operation)**

Repeat Steps 3–6 till all decision plans are retrieved and output the final solution {(*X*_a_*, *Z*_b_*), Y*, h*).

#### 3.6.1. Analysis of Complexity of Algorithm

The analysis of the complexity of computing for the above algorithm is given as follows. The first loop operation of the above algorithm (i.e., logistics authority implicit enumeration loop) requires *n*∗*m* binary decision variables, where *n*, *m* are the length of vector of the 0-1 variable *x*_*a*_^*m*^ and vector of the 0-1 variable *z*_*a*_, respectively. The complexity of computing during this loop is *O*(*mn*).

The second loop operation (logistics operators loop) determines the optimal logistics service frequency and fare for every logistics operator, which is Nash equilibrium game [43, 44]. According to the complexity of computing of Nash equilibrium [45, 46] and the characteristic of our case, we can estimate the complexity of computing approaches *O*(*n*lg*n* + *nm*)^2^, where *n* is the number of logistics operators and *n* is the available combined scheme of the logistics service frequency and fare.

The third loop operation (logistics service path flow assignment loop) aims to obtain the logistics demand *q*_*w*_^(*t*)^ and logistics service route flow assignment *h*_  _^(*t*)^ = {*h*_*wr*_^*m*(*t*)^} with the method of successive average (MSA), in which the complexity of computing is *O*(*n*), where *n* is the number of logistics service route [47–49].

## 4. Illustrative Numerical Example

### 4.1. Input Data

**Figure 5:**
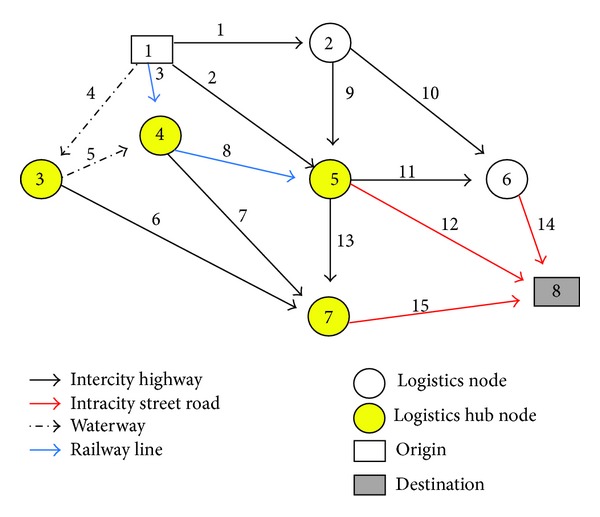
The original logistics service network.

In the following, a numerical example is used to illustrate the applications of the proposed model and solution algorithm. The example regional logistics network is shown in Figure 5. It consists of a logistics demand OD pairs, that is, OD pair 1 (1→8), eight nodes and fifteen links which are denoted by arc* i*  (*i* = 1,2, 3,…, 15). In this figure, nodes 3, 4, 5, and 7 are assumed to be four candidate logistics parks and the maximal number of logistics parks to construct is three.

**Figure 6:**
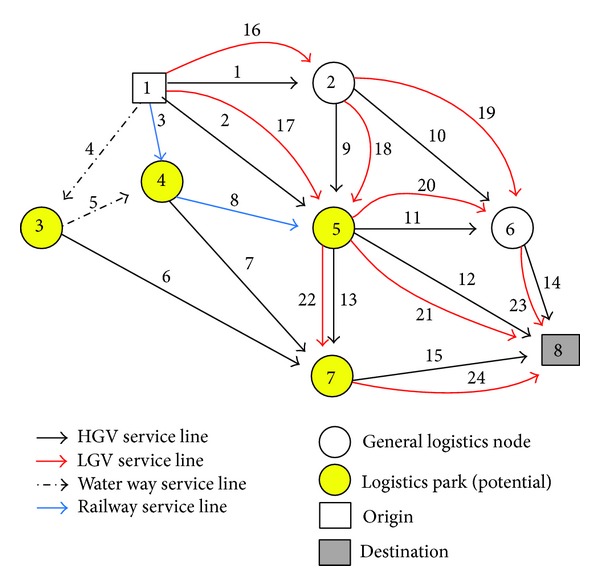
The modified logistics service network.

Figures 5 and 6 show the original and modified logistics service networks, respectively. For some links, different vehicle types are available, and one link that is served by different vehicle types can thus be expanded as different links. For example, link 1 in the original logistics service network (i.e., Figure 5) can be expanded as arcs 1 and 16, as shown in the modified logistics service network (i.e., Figure 6). The basic data for the logistics service network are given in Table 2. The transfer service time and cost are given as shown in Table 3. And the operator parameters on logistics nodes are given as shown in Table 4.

Suppose that the potential demand of logistics OD pair (from node 1 to node 8) is 2000 units per week. The parameters *θ* and *β* are 0.026 and 0.08, respectively. The value of time (VOT) is $20 per hour. The transfer cost and time at the logistics transfer node are shown in Table 3. The other parameters are shown in Table 4. The average emissions of different transportation modes are different. Specifically, the average emissions of HGV, LGV, railway, and waterway are 0.672, 0.865, 0.345, and 0.245 (kg/ton-km), respectively. In the following analysis, unless specifically stated otherwise, these input data are considered as the base case.

### 4.2. Simulation Result

The proposed solution algorithm was coded in Microsoft Visual C++ 6 and run on a laptop Dell N5040 with an Intel Pentium 2.13 GHz CPU and 4.00 GB random-access memory (RAM). And the iterative process for the reference case takes about 283.21 s of CPU time.

Applying the proposed model and solution algorithm to the example network, the results are summarized in Tables 5 and 6. In particular, the following results are worth emphasizing.

For government, the optimal logistics parks locations are parks 4 and 7 and optimal CO_2_ emission charge is 0.3 $/kg. The corresponding customer surplus, producer surplus, and total social welfare are 145831.40, 50555.38, and 80631.02, and unit shipment CO_2_ emission is 65.22 kg/ton. The optimal decision for all logistics operators is given as shown in Table 5. The resultant demand is 1315.47 ton/week according to elastics demand equation (2), of which 987.57 ton/week is allocated to the combined mode network but only 327.43 ton/week to the single mode network. Here, the single mode network means that the logistics service is finished by LGV.

#### 4.2.1. Analysis of Impacts of CO_2_ Emission Charge

**Figure 7:**
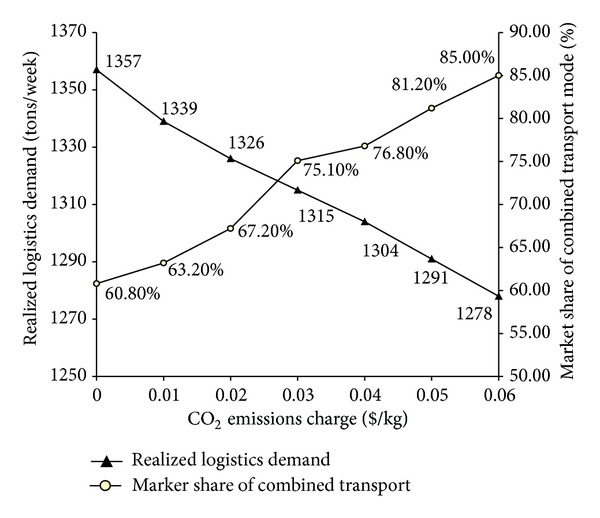
Effects of CO_2_ emission taxes on logistics realized demand.

In the following, we examine the effects of the CO_2_ emission taxes on the realized logistics demand and the market share of the combined mode.  Figure 7 shows that, as the CO_2_ emission taxes increase, the realized logistics demand decreases, whereas the market share of the combined transportation mode increases. This is because the CO_2_ emission tax policy increases the cost of the logistics operator due to an extra payment, which would be transferred to logistics users.

**Figure 8:**
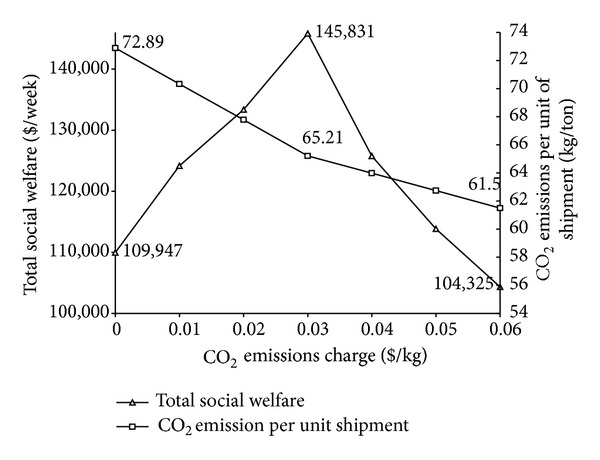
Effects of CO_2_ emission taxes on the total social welfare unit shipment CO_2_ emissions.

Figure 8 further shows the effects of the CO_2_ emission taxes on the logistics network performance in terms of the total social welfare and the CO_2_ emissions per unit of shipment. It can be seen in Figure 8 that the introduction of the CO_2_ emission taxes can lead to a decrease in the CO_2_ emissions per unit of shipment, but the social welfare initially shows a growing trend and later a downward trend. So it is important to set a rational CO_2_ emission charge to keep optimal social welfare and resultant logistics demand.

#### 4.2.2. Analysis of Impacts of Logistics Parks

**Figure 9:**
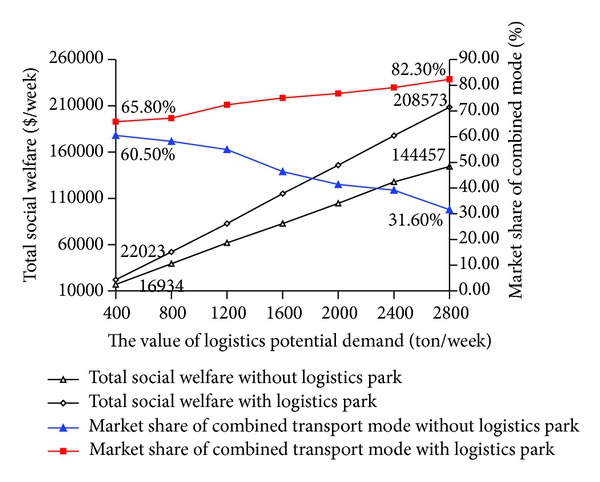
Social welfare and market share of combined mode comparison with constructing logistics parks and without constructing logistics parks.

**Figure 10:**
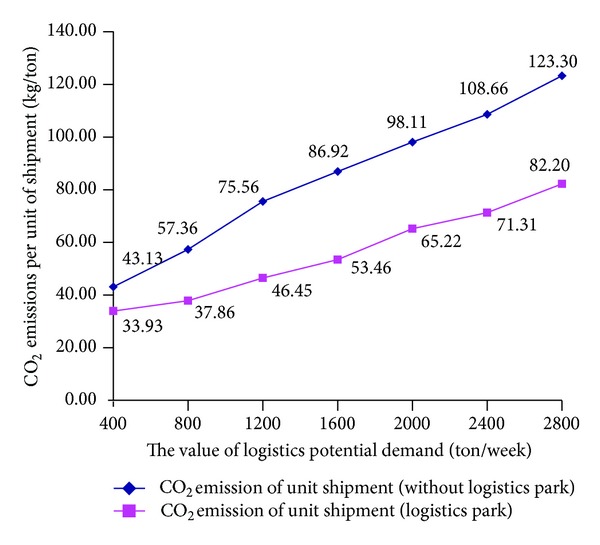
Unit shipment CO_2_ emission comparison with constructing logistics parks and without constructing logistics parks.

In this section, we compare the results with constructing logistics parks and without constructing logistics parks. The detailed analysis results are shown in Figures 9 and 10.

As Figure 9 shows, the market share of combined transport mode increases with the increasing of potential demand under constructing logistics park, while that without logistics park would decreases. The total social welfare with logistics park is more than that without logistics park, and the difference gap becomes larger with the increase of the logistics potential demand.

It can be seen from Figure 10 that the unit shipment CO_2_ emission is lower in the circumstances with logistics parks than that without logistics parks.

From the above analysis, we can get the following conclusions.

1. It is an effective method to decrease CO_2_ emission by combined transport mode.
2. Generally, more social welfare will be obtained by constructing logistics park and unit shipment CO_2_ emission would decrease.
3. Charging CO_2_ emission tax will benefit to decrease unit shipment CO_2_ emission, and it is very important to set rational level to improve the total social welfare.

## 5. Summary

The logistics network is a complex system. There is multilevel decision-making in the system. The logistics authority decides the logistics park location and the number of parks that should be constructed and the carbon price. The logistics operators will decide service charges and frequency with logistic park configuration and carbon emission charge, while the logistics user will choose the path of logistics subject to the price and frequency of upper logistics services. Decisions of different levels are influenced mutually. The decision-making is from top to bottom, and information of the lower level will be fed back to the upper level. The decisions of the upper improve continuously until their objectives are optimal. For such an interaction system, every level's decision will impact the overall system performance and unit shipment CO_2_ emissions.

The proposed model addressed the interaction among the logistics authority, logistics operators, and logistics users and also explicitly incorporated the impacts of the scale economics of the logistics park. The logistics authority (government) optimal decision model is formulated as a 0-1 integer programming problem which can be solved by an implicit enumeration algorithm, while the logistics operators' decision can be depicted as a noncooperative Nash Game model, which is solved by a heuristic search algorithm based on a Lagrangian relaxation approach. A multinomial logit model captures the choice behavior of logistic users. A numerical example is given to validate the optimal mode and its corresponding algorithm. Some interesting insights are obtained from the results of simulation.

The logistics authority should improve and adjust the construction of the logistics network based on the logistics operators and logistics users' feedback information, and the carbon charges should be carried out to build an environment-friendly regional logistics network. The logistics authority should pay attention to the transfer facilities of the logistics park as much as possible to increase the scale of the logistics park and to reduce carbon emissions more. Without high transport time requirements, logistics operators should use a combination of transport modes, which can reduce the carbon emissions of the logistics network.

There are several possible extensions of our model and its algorithm. For example, the more efficient and robust algorithm should be examined. It would be interesting to consider the time-dependent CO_2_ emission charge policy. Finally, it would be interesting to consider our problem in a multiperiod setting. Some of these extensions might be much more complicated and may require approaches which are very different from what is used in this paper.

## Figures and Tables

**Table 1:** Contributions to logistics network design problems.

Modeling approach	Objective			Scale economy	Citation
	Total cost	Total time	Environmental cost		
Hub-Spoke location models	*√*			*√*	O'Kelly [8]; Lin and Chen [9]
	*√*	*√*			Sender and Clausen [10]; Alumur et al. [11]

Network equilibrium models	*√*	*√*			Ham et al. [12]; Yamada et al. [13]; Harker and Friesz [14]; Powell and Sheffi [15]; Crainic et al. [16]
	*√*	*√*			Crainic and Rousseau [17]; Crainic [18]; Yamada et al. [19]; Li et al. [20]
	*√*	*√*	*√*		Bauer et al. [21]
	*√*	*√*	*√*	*√*	This paper

**Table 2:** The basic input data for the logistics service network.

Arc	Mode	Length (km)	Average travel time (hour)	Vehicle capacity (tons/vehicle)	Fleet (vehicles )	Fixed vehicle operator cost ($/vehicle-trip)	Variable costs of unit shipment ($/ton-km)
1	HGV	20	0.33	15	6	8	0.38
2	HGV	40	0.67	15	6	8	0.38
3	Railway	20	0.40	120	1	15	0.35
4	Waterway	30	1.00	150	1	20	0.31
5	Waterway	20	0.67	150	1	20	0.31
6	HGV	50	1.67	150	1	8	0.38
7	HGV	40	0.80	120	1	8	0.35
8	Railway	30	0.60	120	1	15	0.38
9	HGV	30	0.50	15	8	8	0.35
10	HGV	40	0.67	15	8	8	0.38
11	HGV	16	0.27	15	6	8	0.38
12	LGV	15	0.33	3	18	3	0.40
13	HGV	20	0.33	15	6	8	0.38
14	LGV	12	0.27	3	15	3	0.40
15	LGV	10	0.22	3	12	3	0.39
16	LGV	20	0.33	15	6	3	0.39
17	LGV	40	0.67	15	6	3	0.39
18	LGV	30	0.50	15	8	3	0.39
19	LGV	40	0.67	15	8	3	0.39
20	LGV	16	0.27	15	6	3	0.39
21	LGV	15	0.33	3	10	3	0.40
22	LGV	20	0.33	15	6	3	0.40
23	LGV	12	0.27	3	12	3	0.40
24	LGV	10	0.22	3	15	3	0.40

Note. HGV: heavy goods vehicle and LGV: light goods vehicle.

**Table 3:** The transfer time and cost at the general logistics nodes.

Mode	HGV	LGV	Railway	Waterway
HGV	0 (0)	0.5 (1.5)	1.5 (3.6)	2 (4.2)
LGV	0.5 (1.5)	0 (0)	1.1 (3.3)	1.2 (3.9)
Railway	1.5 (3.6)	1.1 (3.3)	0 (0)	1.8 (2.7)
Waterway	2 (4.2)	1.2 (3.9)	1.8 (2.7)	0 (0)

Note. The numbers inside and outside the brackets are transfer cost ($/ton) and transfer time (hour), respectively.

**Table 4:** The operator parameters on logistics node.

	Potential logistics park				General node
Logistics node	3	4	5	7	*N*_*i*_ (*i* = 1, 2, 6, 8)
Economy of scale factor *ρ*	0.9	0.9	0.9	0.9	1.0
Capacity (tons/week)	1200	1000	1000	800	150
Fixed construction cost per unit area of logistics node ($/m^2^)	0.533	0.533	0.533	0.533	0.533
Variable cost per unit of shipment ($/ton)	0.22	0.18	0.2	0.2	0.35

**Table 5:** Optimal decision for all operators on fare and frequency.

Arc	Price ($/ton-km)	Frequency (shifts/week)	Flow (tons/week)	Revenue ($/week)
1	1.01	5	191.06	1129.21
2	0.67	5	450.00	3638.15
3	1.13	7	246.86	4660.87
4	0.59	3	114.04	1541.37
5	0.69	1	33.20	358.95
6	0.39	3	80.85	1014.82
7	0.54	5	68.52	941.37
8	0.57	3	211.54	2815.26
9	0.79	3	114.38	1870.74
10	0.58	2	76.68	1040.07
11	1.09	2	32.64	421.58
12	1.49	7	557.50	9871.02
13	0.73	4	185.78	1806.67
14	1.59	6	109.32	1500.68
15	1.67	6	335.14	4414.36
16	1.38	4	94.79	1050.69
17	0.86	4	207.71	2597.80
18	0.67	3	57.87	658.28
19	0.56	3	36.92	391.26
20	1.00	4	47.50	518.94
21	1.15	7	169.61	1880.68
22	0.84	4	48.48	513.63
23	1.27	6	84.42	789.35
24	1.43	6	48.48	292.23

**Table 6:** Revenue and assignment of the transfer logistics nodes.

Node	Flow (tons/week)	Unit price fare of transfer service ($/ton)	Fixed cost ($/week)	Variable cost ($/ton)	Profit ($/week)
1	0	3.50	0	1.75	0.00
2	0	3.50	0	1.75	0.00
3	0	2.98	0	1.75	0.00
4	33.2	2.63	20	1.75	9.05
5	625.61	2.80	80	1.75	576.89
6	109.32	3.50	20	1.75	171.31
7	335.14	3.50	80	1.75	506.50
8	0	3.50	0	1.75	0
